# Supremum of block entanglement for symmetric Gaussian states

**DOI:** 10.1038/s41598-018-25781-2

**Published:** 2018-05-09

**Authors:** Jhih-Yuan Kao, Chung-Hsien Chou

**Affiliations:** Department of Physics, National Cheng Kung University, Tainan, 701 Taiwan

## Abstract

For a system composed of permutationally symmetric Gaussian modes, by identifying the boundary of valid states and making necessary change of variables, the existence and exact value of the supremum of logarithmic negativity (and negativity likewise) between any two blocks can be shown analytically. Involving only the total number of interchangeable modes and the sizes of respective blocks, this result is general and easy to be applied for such a class of states.

## Introduction

Entanglement, as a distinct characteristic of quantum physics from which many interesting properties stem, such as nonlocality, has been shown to be a potential resource for quantum information^1–10^. Among various quantum systems of interest are continuous variable (CV) systems, of which Gaussian states have been a focus of researches, so has the entanglement and measures of information thereof^11–22^. A Gaussian state is a CV state whose Weyl (or Wigner) characteristic function is Gaussian, which results in a Gaussian Winger quasi-probability distribution^22^. A symmetric Gaussian state is one that is invariant under interchange of modes, which has garnered interests over the years^23–27^.

The search for bounds of entanglement have been conducted for different kinds of systems in terms of various measures, e.g. entanglement of formation^28^ for two-mode Gaussian states^29,30^ and geometric measure of entanglement^31–33^ for symmetric qubits^34–36^.

The system of interest in this work is multiple modes in a symmetric Gaussian state, which is invariant under mode exchanges. We will discuss the bipartite entanglement between two blocks of modes, both of which are parts of the aforementioned modes. We’re going to first talk about some basic properties of Gaussian states, and then of symmetric Gaussian states, the primary subject of this work. Building upon this basis, we will show how to characterize a symmetric Gaussian state with proper variables so that a simple constraint can be established, essential for ascertaining the supremua (least upper bounds) of entanglement, with respect to negativity and logarithmic negativity^37^. Attributes and implications of these suprema will be discussed thereafter.

## Entanglement of Gaussian states

### Covariance matrix, partial transpose and Gaussian states

The covariance matrix of quadrature operators *x*_*i*_ and *p*_*i*_ is composed of variances and covariances of them:

1 $${\sigma }_{ij}=\sigma ({\xi }_{i},{\xi }_{j}),$$

where *σ*(*A*, *B*) = 〈{*A* − 〈*A*〉, *B* − 〈*B*〉}〉/2 denotes the covariance of operators (variables) *A* and *B* (variance if *A* = *B*) and *ξ* = (*x*_1_, *p*_1_, *x*_2_, *p*_2_, …)^T^, the subscript of *x* and *p* for the index of parties. For a continuous variable system, because of the commutation relation [*x*_*i*_, *p*_*j*_] = *iħδ*_*ij*_, the covariance matrix of a valid state should satisfy this condition^38^

2 $$\sigma +i\hslash {\rm{\Omega }}/2\ge 0,$$

where $${\rm{\Omega }}={\rm{diag}}((\begin{array}{cc}0 & 1\\ -\,1 & 0\end{array}),(\begin{array}{cc}0 & 1\\ -\,1 & 0\end{array}),\ldots )$$, which is an alternative form of the commutation relation. By Peres criterion^39^, the partial transpose of a separable state must be positive. Note that *ħ* is kept throughout this paper, so whichever convention is adopted or whichever system is considered the result can be easily adjusted. The transpose of a CV state corresponds to a reversal of momentum, which results in Peres-Horodecki-Simon criterion^39–41^ for separability

3 $$\tilde{\sigma }+i\hslash {\rm{\Omega }}/2\ge 0,$$

where the tilde indicates the reversal of momentum of the transposed party, hence partially transposed. This in general is a necessary condition for separability, but not sufficient. Assuming the first moments are zero, achievable by local unitary operations, a Gaussian state can be totally characterized by its covariance matrix^22,41^:

4 $$W(\xi )={\mathscr{N}}\,\exp \,(-\frac{1}{2}{\xi }^{{\rm{T}}}{\sigma }^{-1}\xi ),$$

where *W* is the Wigner distribution and the normalization factor ${\mathscr{N}}$ depends on *σ* and the number of modes. Thus, the covariance matrix can be thought of as a representation of a Gaussian state, and sometimes we’ll just call the covariance matrix *σ* the “state”.

### Logarithmic negativity

Since logarithmic negativity^37^ measures “how negative” the state is after partial transpose, defined as (a particular base isn’t assigned for flexibility)

5 $${E}_{ {\mathcal L} }\equiv \,\mathrm{log}\,\parallel {\rho }^{{{\rm{T}}}_{A}}{\parallel }_{1},$$

where ${E}_{ {\mathcal L} }$ is logarithmic negativity, $\parallel \,\cdot \,{\parallel }_{1}$ stands for trace norm, and ${\cdot }^{{{\rm{T}}}_{A}}$ refers to partial transpose with respect to party A. Since a two-mode Gaussian state is separable if and only if its partial transpose is positive^41,42^, it’s separable if and only if its logarithmic negativity vanishes. The monotonicity of logarithmic negativity under LOCC^43^ (local operations with classical communication) or a PPT (positive partial transpose) operation was proved by Plenio^44^, so it’s a genuine entanglement monontone (or measure). The logarithmic negativity of a bipartite Gaussian state can be determined by^37,45^

6 $${E}_{ {\mathcal L} }=\,{\rm{\max }}\,(\mathrm{log}\,\frac{\hslash }{2{\tilde{\nu }}^{-}},0),$$

in which ${\tilde{\nu }}^{-}$ is the smaller symplectic eigenvalue of the partially transposed state $\tilde{\sigma }$^45^:

7 $$2{({\tilde{\nu }}^{-})}^{2}=\tilde{{\rm{\Delta }}}-\sqrt{{\tilde{{\rm{\Delta }}}}^{2}-4\,{\rm{\det }}\,\sigma },$$

where Δ is an invariant under local symplectic transformation^45^

8 $${\rm{\Delta }}\equiv {\rm{\det }}\,A+{\rm{\det }}\,B+2\,{\rm{\det }}\,C$$

of the covariance matrix $$\sigma =(\begin{array}{cc}A & C\\ {C}^{{\rm{T}}} & B\end{array})$$, *A*, *B* and *C* being 2 × 2 submatrices, and $\tilde{{\rm{\Delta }}}$ is that of the partially transposed state, $\tilde{\sigma }$. Note ${\rm{\det }}\,\sigma ={\rm{\det }}\,\tilde{\sigma }$.

### Symmetric Gaussian states

Now let’s assume we have *N* symmetric modes in a Gaussian state. The entanglement is totally determined by its covariance matrix, a 2*N* × 2*N* matrix^11,12^:

9 $${\sigma ^{\prime} }_{N}=(\begin{array}{cccc}\alpha  & \beta  & \cdots  & \beta \\ \beta  & \alpha  & \beta  & \vdots \\ \vdots  & \beta  & \ddots  & \beta \\ \beta  & \cdots  & \beta  & \alpha \end{array}),$$

where *α* and *β* are 2 × 2 real and symmetric submatrices, and the subscript *N* is for the total number of modes. By performing *N* appropriate local symplectic transformations on each of the *N* modes, corresponding to *N* local unitary transformations on the density operator, the covariance matrix (9) can always be casted into the “standard form”^12,23,41,45,46^:

10 $${\sigma ^{\prime} }_{N}\to {\sigma }_{N}:\alpha \to {\rm{diag}}(a,a),\beta \to {\rm{diag}}(b,c),$$

and hence irregardless of the original *σ′N*, the standard form *σ*_*N*_ and the parameters thereof, *a*, *b*, and *c* will determine the block entanglement.

## Methods

### Orthogonal transformations for quadrature operators as a canonical transformation

Given *N* symmetric modes at a Gaussian state whose covariance matrix is of the form (9), to scrutinize the entanglement between two blocks that have *n*_1_ and *n*_2_ modes respectively, the standard way is to “unitarily localize”^12,23,25^ these two blocks. However here we would like to treat positions and momenta separately, while still enforcing the commutation relation (canonical conjugation) upon them.

Let’s consider a block with *n* modes, *σ*_*n*_. First, the covariances (and variances) of **x** = (*x*_1_, *x*_2_, …, *x*_*n*_) constitute an “**x**-covariance” matrix, defined as ${\sigma }_{ij}^{{\bf{x}}}\equiv \sigma ({x}_{i},{x}_{j})$, which has the same diagonal elements and the same off-diagonal ones. If we want to diagonalize it, we can utilize the fact that for an *n* × *n* matrix *M*_*ij*_ = *ε* + *δ*_*ij*_(*λ* − *ε*), its eigenvalues are *λ* + (*n* − 1)*ε* and *λ* − *ε*, the latter having (*n* − 1)-fold degeneracy. The eigenvector corresponding to the non-degenerate eigenvalue is (1, 1, …, 1). That is, we can introduce these coordinates:

11 $${\bf{X}}\equiv ({X}_{n}=\frac{1}{\sqrt{n}}\,\sum _{i=1}^{n}\,{x}_{i},{u}_{2},{u}_{3},\ldots ,{u}_{n})=O{\bf{x}},$$

where *O* is an orthogonal matrix, which induces a diagonal **X**-covariance matrix.

In order to maintain the commutation relation, we require the transformation for **p** = (*p*_1_, *p*_2_, …, *p*_*n*_) to be

12 $${\bf{P}}=({P}_{n}=\frac{1}{\sqrt{n}}\,\sum _{i=1}^{n}\,{p}_{i},{{\rm{\Pi }}}_{2},{{\rm{\Pi }}}_{3},\ldots ,{{\rm{\Pi }}}_{n})=O{\bf{p}},$$

where *O* is the same orthogonal matrix as in (11). This transformation can similarly diagonalize the **p**-covariance matrix ${\sigma }_{ij}^{{\bf{p}}}\equiv \sigma ({p}_{i},{p}_{j})$. We can show these transformations imply

13 $$\sigma ({X}_{n},{{\rm{\Pi }}}_{i})=\sigma ({u}_{i},{{\rm{\Pi }}}_{j})=\sigma ({u}_{i},{P}_{n})=0,i\ne j.$$

To prove it, denoting *σ*(*x*_*k*_, *p*_*k*_) = *y* and *σ*(*x*_*k*_, *p*_*l*_) = *z* for *k* ≠ *l*:

14 $$\begin{array}{rcl}\sigma [{({\bf{X}})}_{i},{({\bf{P}})}_{j}] & = & \sum _{k,l}\,{O}_{ik}{O}_{jl}\sigma ({x}_{k},{p}_{l})\\  & = & y\,\sum _{k}\,{O}_{ik}{O}_{jk}+z\,\sum _{k,l\ne k}\,{O}_{ik}{O}_{jl}\\  & = & y{\delta }_{ij}+z\,\sum _{k}\,{O}_{ik}\,\sum _{l\ne k}\,{O}_{jl}\\  & = & y{\delta }_{ij}+z\,\sum _{l}\,{O}_{jl}\,\sum _{k\ne l}\,{O}_{ik}.\end{array}$$

Because the rows of *O* except for the first is orthogonal to (1, 1, …, 1), as explained earlier, either ${\sum }_{k}\,{O}_{ik}={\sum }_{k}\,1\cdot {O}_{ik}=0$ or ${\sum }_{l}\,{O}_{jl}=0$, or both. Hence *σ*[(**X**)_*i*_, (**P**)_*j*_] ≠ 0 only when *i* = *j*. From now on we will ignore the subscript of *u*_*i*_ and Π_*i*_. We can obtain^27^:

15 $$\begin{array}{rcl}\sigma ({X}_{n},{X}_{n}) & = & \sigma ({x}_{i},{x}_{i})+(n-1)\sigma ({x}_{i},{x}_{j}),\\ \sigma ({P}_{n},{P}_{n}) & = & \sigma ({p}_{i},{p}_{i})+(n-1)\sigma ({p}_{i},{p}_{j}),\\ \sigma (u,u) & = & \sigma ({x}_{i},{x}_{i})-\sigma ({x}_{i},{x}_{j}),\\ \sigma ({\rm{\Pi }},{\rm{\Pi }}) & = & \sigma ({p}_{i},{p}_{i})-\sigma ({p}_{i},{p}_{j}),i\ne j,\end{array}$$

of which *σ*(*u*, *u*) *and σ*(Π, Π) *doesn*’*t depend on n at all*. Note *σ*_*n*_ doesn’t need to assume the standard form (10) for the results above to apply. We have effectively found a symplectic transformation that diagonalizes the covariance matrix *σ*_*n*_.

### Unitary localization of blocks

If we have two blocks with *n*_1_ and *n*_2_ modes, employing the same methods, we can not only diagonalize each block, but also “concentrate” all the correlations between these two blocks in $({X}_{{n}_{1}},{P}_{{n}_{1}},{X}_{{n}_{2}},{P}_{{n}_{2}})$. Because *σ*(*u*, *u*) and *σ*(Π, Π) has no dependency on *n*, as shown in (15), whether a particular *σ*(*u*, *u*) or *σ*(Π, Π) “comes from” either block doesn’t matter. The covariances with $({X}_{{n}_{1}},{P}_{{n}_{1}},{X}_{{n}_{2}},{P}_{{n}_{2}})$ must always be zero. Therefore, we’ve essentially “unitarily localized” these two blocks^12,23,25^. Because the block entanglement is equivalent to that between two modes, having a positive partial transpose implies separability and vice versa. Hence, using negativities as the entanglement measure is certified^12,23^. Very recently, a novel approach to the separability problem of Gaussian states was proposed^47^.

To obtain the covariance matrix of the unitarily localized modes, while taking into account these two blocks are part of *N* symmetric modes, we can make use of (15):

16 $${\sigma }_{N:{n}_{1}|{n}_{2}}=(\begin{array}{cc}{\alpha }_{N:{n}_{i}} & {\beta }_{N:{n}_{1}|{n}_{2}}\\ {\beta }_{N:{n}_{1}|{n}_{2}} & {\alpha }_{N:{n}_{i}}\end{array}),$$

where *N*:*n*_1_|*n*_2_ refers to the unitary localizations of *n*_1_ and *n*_2_ modes that are part of *N* modes, likewise for *N*:*n*_*i*_, and

17 $$\begin{array}{rcl}{\alpha }_{N:{n}_{i}} & = & {\rm{diag}}\,[\sigma ({X}_{{n}_{i}},{X}_{{n}_{i}}),\sigma ({P}_{{n}_{i}},{P}_{{n}_{i}})]\\  & = & \frac{1}{N}\,{\rm{diag}}\,[{n}_{i}\sigma ({X}_{N},{X}_{N})+(N-{n}_{i})\sigma (u,u),{n}_{i}\sigma ({P}_{N},{P}_{N})+(N-{n}_{i})\sigma ({\rm{\Pi }},{\rm{\Pi }})],\end{array}$$

18 $$\begin{array}{rcl}{\beta }_{N:{n}_{1}|{n}_{2}} & = & {\rm{diag}}\,[\sigma ({X}_{{n}_{1}},{X}_{{n}_{2}}),\sigma ({P}_{{n}_{1}},{P}_{{n}_{2}})]\\  & = & \frac{\sqrt{{n}_{1}{n}_{2}}}{N}\,{\rm{diag}}\,[\sigma ({X}_{N},{X}_{N})-\sigma (u,u),\sigma ({P}_{N},{P}_{N})-\sigma ({\rm{\Pi }},{\rm{\Pi }})],\end{array}$$

by employing identicalness^27^ and by replacing *σ*(*x*_*i*_, *x*_*i*_) and *σ*(*x*_*i*_, *x*_*j*_) with *σ*(*X*_*N*_, *X*_*N*_) and *σ*(*u*, *u*), and replacing *σ*(*p*_*i*_, *p*_*i*_) and *σ*(*p*_*i*_, *p*_*j*_) with *σ*(*p*_*N*_, *p*_*N*_) and *σ*(Π, Π). (17) and (18) are diagonal by *σ*_*N*_ taking the standard form (10).

### Parameters of a symmetric Gaussian state

The block entanglement of an *N*-mode symmetric Gaussian state can be determined by (*a*, *b*, *c*) of (10); yet according to (17) and (18), it can be achieved also by the following four variances: *σ*(*X*_*N*_, *X*_*N*_), *σ*(*P*_*N*_, *P*_*N*_), *σ*(*u*, *u*) and *σ*(Π, Π), but now with one more variable. To eliminate it, we introduce the ratio:

19 $$r\equiv \sigma ({X}_{N},{X}_{N})/\sigma (u,u)$$

and

20 $${\nu }_{D}\equiv \sqrt{\sigma (u,u)\sigma (\Pi ,\Pi )},{\nu }_{N}\equiv \sqrt{\sigma ({X}_{N},{X}_{N})\sigma ({P}_{N},{P}_{N})}.$$

*ν*_*D*_ and *ν*_*N*_ are actually the symplectic eigenvalues for the symmetric covariance matrix *σ*_*N*_, with *ν*_*D*_ being degenerate, and the *N* of *ν*_*N*_ for its *N*-dependence, c.f. (15). Hence, the formulation from (11) to (20) is just the other side of the coin of the work done by Adesso, Serafini and Illuminati^12,23^, who found the symplectic eigenvalues (20), but here we have one additional parameter (19) that allows us to describe a symmetric Gaussian state with these “global” parameters. Using (15), (*a*, *b*, *c*) of (10) are related to (*ν*_*D*_, *ν*_*N*_, *r*) by, c.f. (9) and (10):

21 $$\begin{array}{rcl}{\rm{\det }}\,\alpha  & = & {a}^{2}=\tfrac{{\nu }_{N}^{2}(N+r-1)+{\nu }_{D}^{2}r(N-1)\,(N+r-1)}{{N}^{2}r}={(\tfrac{\hslash }{2{\mu }_{1}})}^{2}\\ {\rm{\det }}\,\beta  & = & bc=\tfrac{(1-r)\,({\nu }_{D}^{2}r-{\nu }_{N}^{2})}{{N}^{2}r},\\ {\rm{\det }}\,{\sigma }_{2} & = & {\rm{\det }}\,(\begin{array}{cc}\alpha  & \beta \\ \beta  & \alpha \end{array})=({a}^{2}-{b}^{2})\,({a}^{2}-{c}^{2})=\tfrac{{\nu }_{D}^{2}(N+2r-2)\,[2{\nu }_{N}^{2}+{\nu }_{D}^{2}r(N-2)]}{{N}^{2}r}={(\tfrac{\hslash }{2})}^{4}\tfrac{1}{{\mu }_{2}^{2}},\end{array}$$

where *μ*_1_ and *μ*_2_ are the one- and two-mode purities^45^.

The advantage of applying the parameters (*ν*_*D*_, *ν*_*N*_, *r*) over (*a*, *b*, *c*) is that *ν*_*D*_ ≥ *ħ*/2, *ν*_*N*_ ≥ *ħ*/2 and *r* > 0 are necessary conditions for the the state to be valid, i.e. obeying (2), therefore giving us a simpler constraint. To see that those are indeed necessary for obeying (2), note that under the aforementioned coordinate transformation, the covariance matrix is diagonalized as

22 $${\rm{diag}}\,[(\begin{array}{cc}\sigma ({X}_{N},{X}_{N}) & 0\\ 0 & \sigma ({P}_{N},{P}_{N})\end{array}),(\begin{array}{cc}\sigma (u,u) & 0\\ 0 & \sigma ({\rm{\Pi }},{\rm{\Pi }})\end{array}),(\begin{array}{cc}\sigma (u,u) & 0\\ 0 & \sigma ({\rm{\Pi }},{\rm{\Pi }})\end{array}),\ldots ,(\begin{array}{cc}\sigma (u,u) & 0\\ 0 & \sigma ({\rm{\Pi }},{\rm{\Pi }})\end{array})].$$

Therefore each mode has to obey the uncertainty relation, $\sqrt{\sigma (q,q)\sigma (p,p)}\ge \hslash /2$, leading to *ν*_*D*_ ≥ *ħ*/2 and *ν*_*N*_ ≥ *ħ*/2. *r* > 0 simply reflects the fact that each variance is positive, or has the same sign. Were we to define *r* as *σ*(*P*_*N*_, *P*_*N*_)/*σ*(Π, Π), we could obtain the same final result.

Since the ratio *ν*_*N*_/*ν*_*D*_ will prove important later, we replace *ν*_*N*_ with

23 $$\gamma \equiv {\nu }_{N}/{\nu }_{D},$$

which satisfies *γ* ≥ *ħ*/(2*ν*_*D*_) because *ν*_*N*_ = *ν*_*D*_*γ* ≥ *ħ*/2, and we’ll use the three parameters (*ν*_*D*_, *γ*, *r*) from now on, replacing (*a*, *b*, *c*) of (10).

### Block Entanglement for a symmetric Gaussian state

Because the (indexes of the) blocks are interchangeable, the entanglement will depend on the sum and difference of *n*_1_ and *n*_2_:

24 $${n}_{s}\equiv {n}_{1}+{n}_{2}\ge 2,{n}_{d}\equiv |{n}_{1}-{n}_{2}|\ge 0,$$

satisfying *N* ≥ *n*_*s*_ > *n*_*d*_. It’s worth pointing out that only the absolute value of *n*_1_ − *n*_2_ matters, not its sign, as implied by the interchangeability. By (7) the square of the smaller symplectic eigenvalue of ${\tilde{\sigma }}_{N:{n}_{1}|{n}_{2}}$, partial transpose of (16), is a function of (*ν*_*D*_, *γ*, *r*):

25 $${f}_{N:{n}_{s},{n}_{d}}\equiv ({\tilde{{\rm{\Delta }}}}_{N:{n}_{1}|{n}_{2}}-\sqrt{{({\tilde{{\rm{\Delta }}}}_{N:{n}_{1}|{n}_{2}})}^{2}-4\,{\rm{\det }}\,{\sigma }_{N:{n}_{1}|{n}_{2}}})/2,$$

where Δ is defined in (8) and *n*_*s*_, *n*_*d*_ after the colon refers to the mode numbers of the unitarily localized blocks (24). The domain of ${f}_{N:{n}_{s},{n}_{d}}$ is

26 $$\{({\nu }_{D}\ge \hslash /2,\gamma ,r > 0):{\nu }_{D}\gamma \ge \hslash /2\},$$

which is defined by the set of all valid Gaussian symmetric states. By (6) the logarithmic negativity between two blocks is

27 $${E}_{ {\mathcal L} }^{N:{n}_{1}|{n}_{2}}={E}_{ {\mathcal L} }^{N:{n}_{s},{n}_{d}}=\frac{1}{2}\,\mathrm{log}\,{\rm{\max }}(\frac{{\hslash }^{2}}{4{f}_{N:{n}_{s},{n}_{d}}},1);{n}_{s}={n}_{1}+{n}_{2},{n}_{d}=|{n}_{1}-{n}_{2}|.$$

Let’s summarize what we have done so far: Any symmetric Gaussian state of the form (9) can be turned into the “standard form” by local unitary operations, with parameters given in (10). After orthogonal transformations on the quadrature operators and with a proper choice of parameters, the logarithmic negativity between two blocks (27) becomes a function with a clear domain (26). In the following subsection, we will show how this new parametrization will help us to find the suprema of negativities.

### Search for the supremum/infimum

By (27) the supremum of ${E}_{ {\mathcal L} }$, if it exists, corresponds to the infimum (greatest lower bound) of ${f}_{N:{n}_{s},{n}_{d}}$, ${\rm{\inf }}\,{f}_{N:{n}_{s},{n}_{d}}$. Hence our goal is to find this infimum. Let’s start with

28 $$\begin{array}{rcl}{f}_{N:{n}_{s},{n}_{d}}({\nu }_{D},\gamma ,r) & = & \frac{{\nu }_{D}^{2}}{2{N}^{2}r}\{(N{n}_{s}-{n}_{d}^{2})\,{r}^{2}+[2{N}^{2}-2N{n}_{s}+{n}_{d}^{2}(1+{\gamma }^{2})]\,r\\  &  & +(N{n}_{s}-{n}_{d}^{2})\,{\gamma }^{2}-\{{(N{n}_{s}-{n}_{d})}^{2}\,{r}^{4}-2{n}_{d}^{2}[2{N}^{2}+{n}_{d}^{2}(1+{\gamma }^{2})\\  &  & -N{n}_{s}(3+{\gamma }^{2})]\,{r}^{3}+[{N}^{2}\,(4{n}_{d}^{2}(1+{\gamma }^{2})-2{n}_{s}^{2}{\gamma }^{2})\\  &  & +{n}_{d}^{4}\,({\gamma }^{4}+4{\gamma }^{2}+1)-4N{n}_{s}{n}_{d}^{2}\,(1+2{\gamma }^{2})]\,{r}^{2}\\  &  & -2{n}_{d}^{2}\,[2{N}^{2}+{n}_{d}^{2}\,(1+{\gamma }^{2})-N{n}_{s}\,(3+{\gamma }^{2})]\,{\gamma }^{2}r+{(N{n}_{s}-{n}_{d}^{2})}^{2}\,{\gamma }^{4}{\}}^{1/2}\},\end{array}$$

continuous everywhere on the domain (26). With three variables, first let’s vary *r* only while keeping *ν*_*D*_ and *γ* fixed, by defining

29 $${F}_{{\nu }_{D},\gamma }^{N:{n}_{s},{n}_{d}}(r)\equiv {f}_{N:{n}_{s},{n}_{d}}({\nu }_{D},\gamma ,r)$$

as a function of *r* only. *F* is of this form (henceforth we’ll ignore the superscripts and subscripts of *f* and *F*):

30 $$F(r)=\frac{{\nu }_{D}^{2}}{{N}^{2}}\frac{p(r)-\sqrt{h(r)}}{r},$$

where *p* and *h* are polynomials of second and forth orders respectively, whose coefficients are made up of (*γ*, *N*, *n*_*s*_, *n*_*d*_), *without ν*_*D*_. Because *F*(*r*) is a differentiable function of *r* ∈ (0, ∞), the global minimum or infimum lies on the boundary or where the derivative vanishes. Let us first examine the behavior of *F*(*r*) at the boundary.

#### Boundary

Using L’Hôpital’s rule, the limits of *F* as *r* approaches the boundary 0 and ∞ are

31 $$\mathop{\mathrm{lim}}\limits_{r\to 0}\,F(r)=\mathop{\mathrm{lim}}\limits_{r\to \infty }\,F(r)={\nu }_{D}^{2}\frac{N{n}_{s}-{n}_{s}^{2}}{N{n}_{s}-{n}_{d}^{2}},$$

*independent of γ*, or *ν*_*N*_. Note it’s nonnegative and smaller than ${\nu }_{D}^{2}$ because *N* ≥ *n*_*s*_ > *n*_*d*_.

#### Critical point

Next, we shall investigate where *F*′(*r*) = 0. By applying the lemma that can be found in the supplementary material, the critical point is at *r* = *γ*. In addition, *F*(0) = *F*(∞) (which will denote ${\mathrm{lim}}_{r\to 0}\,F(r)$ and ${\mathrm{lim}}_{r\to \infty }\,F(r)$ hereafter), differentiability of *F*(*r*), and the existence of only one critical point imply that *F*(*γ*) is either a global maximum or minimum, and that *F*(0) = *F*(∞) is either the supremum or infimum; that is, the smaller one of *F*(0) = *F*(∞) and *F*(*γ*) is the infimum of *F*.

#### Value at the critical point

However, instead of directly comparing *F*(0) = *F*(∞) and *F*(*γ*), let’s see how small *F*(*γ*) can be, because if *F*(*γ*) ≥ *ħ*^2^/4, then the entanglement measure would be zero by (27); namely, this point is of no interest to us at all! By (25),

32 $$\begin{array}{rcl}F(\gamma ) & = & \frac{{\nu }_{D}^{2}}{2{N}^{2}}[2{N}^{2}+2N{n}_{s}(\gamma -1)+{n}_{d}^{2}{(\gamma -1)}^{2}\\  &  & -{n}_{d}|\gamma -1|\sqrt{4{N}^{2}+4N{n}_{s}(\gamma -1)+{n}_{d}^{2}{(\gamma -1)}^{2}}].\end{array}$$

Let’s define

33 $$g(\gamma )\equiv F(r=\gamma )$$

*as a continuous function of γ*, and

34 $$q\equiv \hslash /(2{\nu }_{D})\in (0,1].$$

Because *ν*_*N*_ = *ν*_*D*_*γ* ≥ *ħ*/2 by (23), *γ* ≥ *q*. We consider two cases separately for *g*(*γ*), i.e.*γ* > 1 and 1 ≥ *γ* ≥ *q*:

1. *γ* > 1: Defining *x* ≡ *γ* − 1 > 0, we try to solve *g*(*γ* = *x* + 1) < *ħ*^2^/4 with the constraint *q* ≤ 1. This leads to a quadratic inequality *c*_2_*x*^2^ + *c*_1_*x* + *c*_0_ < 0, where *c*_2_ > 0, *c*_1_ ≥ 0 and *c*_0_ ≥ 0. Hence, it has no nonnegative solutions. More details can be found in the supplementary material.
2. 1 ≥ *γ* ≥ *q*: Defining *x* ≡ 1 − *γ* ∈ [0, 1 − *q*], the solution of *g*(*γ* = 1 − *x*) < *ħ*^2^/4 is the interval

35 $$(\frac{N(1-{q}^{2})}{{n}_{s}+{n}_{d}q},\frac{N(1-{q}^{2})}{{n}_{s}-{n}_{d}q}).$$

Since the greatest lower bound of the interval above

36 $$\frac{N(1-{q}^{2})}{{n}_{s}+{n}_{d}q}=\frac{N(1+q)}{{n}_{s}+{n}_{d}q}(1-q)\ge 1-q$$

by *N* ≥ *n*_*s*_ > *n*_*d*_ ≥ 0 and *q* > 0, no solution exists for *x* ∈ [0, 1 − *q*].

Therefore, *g*(*γ*) = *F*(*γ*) ≥ *ħ*^2^/4 on the domain *γ* ∈ [*q*, ∞), so when inf *F* is smaller than *ħ*^2^/4, inf *F* is definitely *F*(0) = *F*(∞).

### Data availability

No data was generated or analyzed for this study.

## Results

Thanks to the continuity of *f*, (28), $\forall \varepsilon  > 0$ as $({\nu }_{D},\gamma )\to ({\nu }_{D}^{^{\prime} },\gamma ^{\prime} )$

37 $$f({\nu }_{D},\gamma ,\varepsilon )\to f({\nu ^{\prime} }_{D},\gamma ^{\prime} ,\varepsilon ),$$

so as $$({\nu }_{D},\gamma ,r)\to ({\nu ^{\prime} }_{D},\gamma ^{\prime} ,0)$$

38 $$f({\nu }_{D},\gamma ,r)\to f({\nu ^{\prime} }_{D},\gamma ^{\prime} ,r)\to \mathop{\mathrm{lim}}\limits_{r\to 0}\,{F}_{{\nu ^{\prime} }_{D},\gamma ^{\prime} }(r),$$

i.e. the limit as *r* → 0 is identical for *f* and *F*, same if *r* → ∞. Hence, when inf *F* < *ħ*^2^/4, *F*(0) = *F*(∞) is the infimum of *f* at a fixed *ν*_*D*_, for *F*(0) = *F*(∞) is independent of *γ*, or *ν*_*N*_. From (27) and (31), we have:

### *Proposition 1*:

*For N modes in a symmetric Gaussian state at a given ν*_*D*_, *the degenerate symplectic eigenvalue* (20), *the supremum of logarithmic negativity between two blocks containing n*_1_ *and n*_2_ *modes is*

39 $${\rm{\sup }}\,{E}_{ {\mathcal L} :{\nu }_{D}}^{N:{n}_{1}|{n}_{2}}={E}_{ {\mathcal L} :{\nu }_{D}}^{N:{n}_{s},{n}_{d}}=\,{\rm{\max }}\,[\frac{1}{2}\,\mathrm{log}\,\frac{{\hslash }^{2}}{4{\nu }_{D}^{2}}(1+\frac{{n}_{s}^{2}-{n}_{d}^{2}}{{n}_{s}(N-{n}_{s})}),0];{n}_{s}={n}_{1}+{n}_{2},{n}_{d}=|{n}_{1}-{n}_{2}|.$$

Likewise, because the infimum of *f*(*ν*_*D*_, *γ*, *r*) over all (*ν*_*D*_, *γ*, *r*) is the infimum of $f({\nu }_{D},\gamma ,0)={F}_{{\nu }_{D},\gamma }(0)$ over all (*ν*_*D*_, *γ*) and because *ν*_*D*_ ≥ *ħ*/2, we obtain:

### *Proposition 2*:

*Using the same notations as in proposition 1*, *for all symmetric Gaussian states*:

40 $${\rm{\sup }}\,{E}_{ {\mathcal L} }^{N:{n}_{s},{n}_{d}}=\frac{1}{2}\,\mathrm{log}\,(1+\frac{{n}_{s}^{2}-{n}_{d}^{2}}{{n}_{s}(N-{n}_{s})}).$$

Note (40) is always positive, while (39) is nonnegative. Similar results apply to negativity ${E}_{{\mathscr{N}}}$^37^ by the relation ${E}_{{\mathscr{N}}}=({t}^{{E}_{ {\mathcal L} }}-1)/2$, assuming the base is *t*. Namely,

### *Proposition 3*:

*Following the notations like proposition 1*, *for all symmetric Gaussian states the negativity between two blocks has a supremum*:

41 $${\rm{\sup }}\,{E}_{{\mathscr{N}}}^{N:{n}_{s},{n}_{d}}=\frac{1}{2}\,[{(1+\frac{{n}_{s}^{2}-{n}_{d}^{2}}{{n}_{s}(N-{n}_{s})})}^{1/2}-1].$$

*At a given ν*_*D*_:

42 $${\rm{\sup }}\,{E}_{{\mathscr{N}}:{\nu }_{D}}^{N:{n}_{s},{n}_{d}}=\,{\rm{\max }}\,\{\frac{1}{2}[\frac{\hslash }{2{\nu }_{D}}{(1+\frac{{n}_{s}^{2}-{n}_{d}^{2}}{{n}_{s}(N-{n}_{s})})}^{1/2}-1],0\}.$$

Furthermore, by intermediate value theorem^48,49^, with two blocks at given (*N*, *n*_*s*_, *n*_*d*_) and perhaps *ν*_*D*_, for all elements in the following interval:

43 $$[0,{\rm{\sup }}\,E),$$

where sup *E* is (39), (40), (41) or (42) depending on whether *ν*_*D*_ is fixed and which measure is adopted, we can always find a corresponding symmetric Gaussian state, unless sup *E* = 0 (which can only happen with fixed *ν*_*D*_, (39) and (42)), in which case the entanglement measure can only be zero. In other words, the mapping from a symmetric Gaussian state to the interval (43) is surjective (but not injective). Note these suprema aren’t maxima, so the interval (43) is not closed.

## Discussions

### Boundedness and block sizes

Suppose we have two blocks, with *n*_1_ and *n*_2_ modes, from *N* symmetric modes. As *N* increases, the supremum of bipartite entanglement between them decreases, unless *n*_1_ + *n*_2_ = *N*. What is the reason behind? By increasing the total number of interchangeable modes, we actually impose a stronger constraint on individual modes, i.e. (*a*, *b*, *c*) of (10). This should be clearer with the help of (15): In terms of the parameters (*ν*_*D*_, *ν*_*N*_, *r*) as defined by (19) and (20), which is interchangeable with (*a*, *b*, *c*), we can find as *r* → 0 or *r* → ∞

44 $$\sigma ({X}_{N^{\prime} },{X}_{N^{\prime} })\sigma ({P}_{N^{\prime} },{P}_{N^{\prime} })\to \{\begin{array}{cc}-\infty  & {\rm{if}}\,N^{\prime}  > N\\ {\nu }_{N} & {\rm{if}}\,N^{\prime} =N\\ \infty  & {\rm{if}}\,N^{\prime}  < N\end{array}.$$

This implies that at both limits the symmetric state remains legitimate with *N* total modes or less, but becomes invalid if there are *N*′ > *N* symmetric modes in total, violating (2); hence we’re left with “less” choices (still infinite) of (*a*, *b*, *c*) as *N* grows. Please heed that with the same “local state”, or (*a*, *b*, *c*), the entanglement between two blocks of given sizes should be the same, irregardless of how large *N* is: What *N* does is restricting the choice of “local states”, which in turn restricts entanglement. A valid local state for *N* = *N*_1_ may be invalid for *N* = *N*_2_ > *N*_1_.

In the supplementary material, we prove that for all symmetric Gaussian states, at fixed *n*_*s*_ and *N* a larger *n*_*d*_ decreases the negativities of block entanglement, which was observed by Serafini *et al*.^12^ The suprema (39)~(42) comply with this phenomenon as well: As the blocks become more equal, they become more entangled. This suggests an optimal strategy of “gathering” entanglement^12^.

At the same state, the block entanglement between subsystems should be smaller than or equal to that between the systems. This is reflected by the fact that a larger *n*_*s*_ implies larger suprema, which can be checked by partially differentiating them. Furthermore, we can replace *n*_*d*_ and *n*_*s*_ with *n*_1_ and *n*_2_, and find that these suprema do increase by adding more modes to either block.

### Monogamy of entanglement and multipartite entanglement

Monogamy of entanglement^50,51^, describing the phenomenon that entanglement between multiple parties can’t be shared freely, can be embodied by an inequality first introduced by Coffman, Kundu, and Wooters (CKW)^50,52^:

45 $${E}^{{p}_{1}|({p}_{2},\ldots ,{p}_{N})}\ge \sum _{i=1}^{N}\,{E}^{{p}_{1}|{p}_{N}},$$

where *p*_*i*_ denotes the *i*-th party, and ${E}^{{p}_{i}|{p}_{j}}$ is the bipartite entanglement between party *i* and party *j*. In the tripartite case, the difference between the left and right hand sides of the inequality above is the residual entanglement, characterizing the tripartite entanglement^14^. However, not all genuine entanglement measures obey this inequality; instead, a class of entanglement measures has been chosen to satisfy this condition, such as tangle^50^ and cotangle^14,53^. A stronger form of entanglement monogamy was also proposed^25,54^:

46 $${E}^{{p}_{1}|({p}_{2},\ldots ,{p}_{N})}=\sum _{j=2}^{N}\,{E}^{{p}_{1}|{p}_{j}}+\sum _{k > j=2}^{N}\,{E}^{{p}_{1}|{p}_{j}|{p}_{k}}+\cdots +{E}^{\underline{{p}_{1}}|{p}_{2}|\ldots |{p}_{N}},$$

where the second and latter terms are all multipartite entanglements with more than two parties. The genuine residual *N*-partite entanglement ${E}^{{p}_{1}|{p}_{2}|\ldots |{p}_{N}}$ can be found by minimizing ${E}^{\underline{{p}_{1}}|{p}_{2}|\ldots |{p}_{N}}$, choosing the appropriate probing party $\underline{{p}_{i}}$^25,54^. In principle an *N*-partite entanglement can be attained by iteration, with the information of bipartite entanglements^25,54^.

Even though negativity and logarithmic negativity, the focus of our present paper, in general don’t obey CKW inequality^14^, our results show some interesting aspects regarding Gaussian multipartite entanglement: The bipartite block entanglement, with respect to negativities, becomes unbounded when *n*_*s*_ = *N*, bounded otherwise. If we have *m* blocks from *N* symmetric modes, with ${n}_{1}+\cdots +{n}_{m} < N$, then since all bipartite entanglement between them is bounded, (46) implies that the *m*-partite entanglement among these *m* blocks is also bounded. On the other hand, if ${n}_{1}+\cdots +{n}_{m}=N$, because the bipartite entanglement between one and the remaining parties is unbounded, we expect the multipartite entanglement involving all of them is unbounded as well. In short, if the blocks fill all the symmetric modes, the multipartite entanglement is expected to be unbounded; bounded if not.

A few more words can be also said on the boundedness: Because when *n*_1_ + *n*_2_ = *N* the entanglement is unbounded, that the maximal entanglement between blocks of given sizes decreases with increasing *N* isn’t due to a limited amount of entanglement distributed to more parties (by (45), the monogamy of entanglement), leading to every party gaining less: There’s (potentially) infinite entanglement to begin with.

Rigorously speaking, this problem should be treated with a suitable measure that obeys (45), like cotangle. However, this is beyond our current scope, and we hope this primitive discussion can inspire future works.

### Available entanglement

Because logarithm is monotonic, how sup *E* behaves depends on (c.f. (39)~(42))

47 $$K(N,{n}_{s},{n}_{d})\equiv \frac{{n}_{s}^{2}-{n}_{d}^{2}}{{n}_{s}(N-{n}_{s})}.$$

For *n*_*s*_ < *N*, the greatest supremum can be achieved when *n*_*s*_ = *N* − 1, with the corresponding *K* being

48 $$K=\{\begin{array}{ll}N-1 & {\rm{for}}\,{\rm{odd}}\,N\,{\rm{and}}\,{n}_{d}=0\\ N-1-1/(N-1) & {\rm{for}}\,{\rm{even}}\,N\,{\rm{and}}\,{n}_{d}=1\end{array},$$

which clearly becomes larger as *N* increases. Therefore, excluding the case *n*_*s*_ = *N*, as the total number of symmetric modes *N* increases, we can actually have more entanglement at out disposal.

By (39) and (42) at a given *ν*_*D*_ no entanglement can exist between any two blocks if

49 $$K(N,{n}_{s},{n}_{d})+1\le \frac{4{\nu }_{D}^{2}}{{\hslash }^{2}},$$

so by (48) when

50 $$\frac{4{\nu }_{D}^{2}}{{\hslash }^{2}}\ge \{\begin{array}{ll}N & {\rm{for}}\,{\rm{odd}}\,N\\ N-1/(N-1) & {\rm{for}}\,{\rm{even}}\,N\end{array},$$

two blocks with *n*_*s*_ < *N* can only be separable; in other words for symmetric modes with *ν*_*D*_ satisfying the condition above any two blocks are separable unless *n*_*s*_ = *N*.

### Purities

If we can gain information of *ν*_*D*_, then it’s possible to lower the supremum, i.e. using (39) instead of (40). A symmetric Gaussian pure state has *ν*_*D*_ = *ν*_*N*_ = *ħ*/2, and for bisymmetric^12^ or multisymmetric Gaussian states^25^ composed of several clusters of symmetric modes *ν*_*D*_ = *ħ*/2, so for those states the upper bound cannot be lowered. Because the global purity *μ* of symmetric Gaussian modes is^23,45^

51 $$\mu =\frac{1}{{\nu }_{N}{\nu }_{D}^{N-1}}{(\frac{\hslash }{2})}^{N}=\frac{1}{\gamma }{(\frac{\hslash }{2{\nu }_{D}})}^{N},$$

which fixes the parameter *γ*. However, since the the suprema lie on the boundary *r* → 0 and *r* → ∞, *γ* doesn’t really play a role in determining the values of the suprema. Hence if the global purity is known, which even though can “shrink” the domain, the suprema stay the same.

As a comparison, Adesso *et al*.^11^ found that the entanglement between two modes of a Gaussian state is bounded from below and above if the global (two-mode) and marginal (one-mode) purities are known. However, were we to ascertain the bounds imposed by purities of multiple modes (e.g. global purity) and of one mode for symmetric Gaussian states, it either would constitute a complicated constraint of the domain for the current parameters in use, i.e. (*ν*_*D*_, *γ*, *r*) or (*a*, *b*, *c*) of (10), or while reduces it to a one-variable problem, would require a suitable parameter and technique to find the bounds, and hence not the subject of our work at this stage.

### Two modes and approximation

Next we consider the entanglement between two modes as a special case. When *n*_1_ = *n*_2_ = 1 (40) becomes

52 $${\rm{\sup }}\,{E}_{ {\mathcal L} }^{N:1|1}=\frac{1}{2}\,\mathrm{log}\,(1+\frac{2}{N-2}),$$

as obtained in our previous work^27^, which was proved for a bisymmetric^12^ Gaussian pure state under the assumption that the coefficients of the wave function are real, so in the present work we provide a complete proof and a more general result. Taking the base to be *e*, it has an approximation 1/*N* as $N\gg 1$.

More generally, when $N\gg {n}_{s}$ (and thus $N\gg {n}_{d}$) (40) approximates

53 $${\rm{\sup }}\,{E}_{ {\mathcal L} }^{N:{n}_{s},{n}_{d}}\simeq \frac{{n}_{s}^{2}-{n}_{d}^{2}}{2{n}_{s}N}\simeq \frac{{n}_{s}}{2N},$$

where the second approximation can be made if ${n}_{d}\ll {n}_{s}$ or *n*_*d*_ = 0. By the same token, we acquire ${\rm{\sup }}\,{E}_{{\mathscr{N}}}^{N:{n}_{1}|{n}_{2}}\simeq ({n}_{s}^{2}-{n}_{d}^{2})/(4{n}_{s}N)$, and ${\rm{\sup }}\,{E}_{{\mathscr{N}}}^{N:{n}_{1}|{n}_{2}}\simeq {n}_{s}/(4N)$ when ${n}_{d}\ll {n}_{s}$. Here scaling with respect to *N* and *n*_*s*_ is demonstrated in an explicit manner: proportional to *n*_*s*_ and inversely proportional to *N* when $N\gg {n}_{s}$.

## Conclusion

We have shown the least upper bounds of block entanglement for Gaussian state with *N*-symmetric modes, in terms of logarithmic negativity and negativity, (40) and (41); if *ν*_*D*_ of (20), the degenerate symplectic eigenvalue can be known, (39) and (42) for tighter bounds. Such upper bounds originates from the symmetry of the state, and the basic requirement that the state obey the uncertainty relation, satisfying (2). These two conditions together impose a constraint on the modes, which becomes more stringent for each individual mode as the total number of modes *N* increases; that is, with “local” parameters like (*a*, *b*, *c*) of (10) to describe a symmetric Gaussian state, the domain shrinks as a result of increasing *N*.

Despite the dwindling domain of local parameters, the possibility for the blocks to swell even more, i.e. larger *n*_*s*_ = *n*_1_ + *n*_2_ can not only compensate for that, but the achievable maximal block entanglement, or unitarily localizable entanglement can increases as a result of increasing total modes, (48), at the expense of entanglement between blocks of fixed sizes, say *n*_1_ = *n*_2_ = 1, c.f. some previous works^12,23^. Yet at whichever *N*, for two blocks with *n*_1_ + *n*_2_ = *N*, there exists no upper bound for their block entanglement. In other words, the unitarily localizable entanglement will be wasted if it’s not gathered across all the symmetric modes, but if the localization can be done in full, then the total number of symmetric modes makes little difference, in terms of the amount of available resource that is entanglement. Moreover, by the “strong” monogamy of entanglement, the same can be anticipated for *m*-partite entanglement, i.e. whether it’s bounded depends on if ${n}_{1}+{n}_{2}+\cdots +{n}_{m}=N$.

Gaussian states, as a subset of states of an infinite-dimensional space, have an unbounded entanglement (with respect to measures like negativities) if there’s no constraint put on them. In this regard, symmetry under mode swapping is a very strong condition, which renders the entanglement bounded. Ascertaining the exact bound was then made possible by choosing global parameters with a clear boundary, (26). The method employed in this work may be helpful in other related issues, where (multi)symmetric Gaussian states are the subject.

## Electronic supplementary material

Supplementary material
