# Supplementary material for: Supremum of block entanglement for symmetric Gaussian states
Source: Sci Rep. 2018 May 9;8:7394. doi: 10.1038/s41598-018-25781-2 (PMC5943598; doi:10.1038/s41598-018-25781-2)
Supplement: Supplementary file 1 — Supplementary material [file 41598_2018_25781_MOESM1_ESM.pdf]

Supremum of block entanglement for symmetric Gaussian states: Supplementary material

Jhih-Yuan Kao and Chung-Hsien Chou
Department of physics, National Cheng Kung University, Tainan 70101, Taiwan

When referring to equations inside this supplement, it's colored; when referring to equations from the main article, it is uncolored and with an additional "eq."

1 The critical point

Lemma

For a function

$$y(x) = \frac{p(x) - \sqrt{h(x)}}{x}, \tag{1}$$

where p and h are second- and fourth-order polynomials:

$$p(x) = \sum_{i=0}^{2} a_i x^i,\ h(x) = \sum_{i=0}^{4} b_i x^i, \tag{2}$$

if not only $\lim_{x\to 0} y(x)$ and $\lim_{x\to\infty} y(x)$ exist but also

$$\lim_{x\to 0} y(x) = \lim_{x\to\infty} y(x), \tag{3}$$

then the solutions of $y'(x) = 0$ are $\pm\sqrt{a_0/a_2}$.

Proof

The existence of $\lim_{x\to 0} y(x)$ and $\lim_{x\to\infty} y(x)$ implies

$$a_0 = \sqrt{b_0},\ a_2 = \sqrt{b_4}. \tag{4}$$

Applying L'Hôpital's rule again, the equivalence of these limits suggests

$$a_1 - \frac{b_1}{2\sqrt{b_0}} = a_1 - \frac{b_3}{2\sqrt{b_4}} \Rightarrow \frac{b_1}{\sqrt{b_0}} = \frac{b_3}{\sqrt{b_4}}. \tag{5}$$

$y'(x) = 0$ induces

$$4h(p'x - p)^2 - (h'x - 2h)^2 = 0. \tag{6}$$

With (4) and (5), we obtain

$$x^2(a_2 x^2 - a_0)^2 = 0, \tag{7}$$

whose roots are $\pm\sqrt{a_0/a_2}$. □

Critical point

The function F, eq. (28), conforms to the form (1). Because $\lim_{r\to 0} F(r) = \lim_{r\to\infty} F(r)$, eq. (31), it satisfies the condition (3). The square root of the ratio is exactly γ, c.f. eq. (28), so the critical point is at $r = \gamma$.

2 The quadratic inequality

By $q \equiv \hbar/(2\nu_D) \in (0,1]$, eq. (34), $g(\gamma = x+1) < \hbar^2/4$, see eqs. (32) and (33), becomes

$$\frac{2N^2 + 2xNn_s + xn_d\left(xn_d - \sqrt{n_d^2x^2 + 4N(N+n_sx)}\right)}{2N^2} < \frac{\hbar^2}{4\nu_D} = q^2, \tag{8}$$

which is equivalent to

$$2N^2(1-q^2) + n_d^2x^2 + 2Nn_sx < n_dx\sqrt{n_d^2x^2 + 4N(N+n_sx)}. \tag{9}$$

Since both sides are positive, for $q \in (0,1]$, this leads to

$$\left(2N^2 + n_d^2x^2 + 2Nn_sx - 2N^2q^2\right)^2 < (n_dx)^2\left[n_d^2x^2 + 4N(N+n_sx)\right],$$

$$\Rightarrow N^2(n_s^2 - n_d^2q^2)x^2 + 2N^3n_s(1-q^2)x + N^4(1-q^2)^2 = c_2x^2 + c_1x + c_0 < 0. \tag{10}$$

By $n_s > n_d$, eq. (24), and $q \in (0,1]$, we obtain $c_2 > 0$, $c_1 \geq 0$ and $c_0 \geq 0$. Following pretty much the same procedure can give us the quadratic inequality for $g(\gamma = 1-x) < \hbar^2/4$.

3 Decrease of entanglement with increasing n_d

Here we provide the proof that for all symmetric Gaussian states, with n_s modes of N total modes, the more even the partition is (less n_d), the greater the negativities are, i.e. stronger entanglement. Please note this is a general result, not only limited to the suprema.

The first thing we need to know is that for the function $F(r)$, eqs. (28)∼(30), because $F(\gamma) \geq \hbar^2/4$, there's a region around γ where F is always larger than or equal to $\hbar^2/4$, irregardless of the values of other parameters, so in this region the entanglement vanishes, eq. (27). Since $\nu_D \geq \hbar/2$, if $F(r) \geq \nu_D^2$, then $F(r) \geq \hbar^2/4$. Let's first try to solve $F(r) = \nu_D^2$. It can be easily found out the roots are $r = 1$ or $r = \gamma^2$. As shown in the main article, $F(\gamma) \geq \hbar^2/4$, and because $F(\gamma)$ is the global maximum,[1] $F(r) \geq \hbar^2/4$ between[2] $r = 1$ and $r = \gamma^2$, that is, we're only interested in this subset of positive r[3]:

$$S \equiv \{r : (r-1)(r-\gamma^2) > 0\}. \tag{11}$$

Taking F, or f, eq. (28), as a function of n_d, it takes this form

$$\frac{\nu_D^2}{2N^2r}\left(a_2n_d^2 + a_0 - \sqrt{b_4n_d^4 + b_2n_d^2 + b_0}\right), \text{ with } b_4 = a_2^2, \text{ and } b_2 = a_2a_0. \tag{12}$$

Partially differentiating it with respect to n_d:

$$\frac{\partial f}{\partial n_d} = \frac{\nu_D^2 n_d}{2N^2r}\left(2a_2 - \frac{2b_4n_d^2 + b_2}{\sqrt{b_4n_d^4 + b_2n_d^2 + b_0}}\right). \tag{13}$$

We'd like to see whether $\partial f/\partial n_d$ is always larger than zero when $f < \hbar^2/4$, which would imply

$$\frac{\partial E^{N:n_s,n_d}}{\partial n_d} < 0 \tag{14}$$

for negativities. Because $f < \hbar^2/4$ only when $r \in S$ (11) we only need to consider this subset S. Since

$$a_2 = -(r-1)(r-\gamma^2), \tag{15}$$

a_2 is always negative for $r \in S$. On the other hand, we can find

$$2b_4n_d^2 + b_2 = -2(r-1)(r-\gamma^2)\left[2rN(N-n_s) + (Nn_s - n_d^2)(r^2+\gamma^2) + n_d^2r(1+\gamma^2)\right] < 0, \tag{16}$$

[1] If it's the global minimum, then $F(r) \geq \hbar^2/4$ at all r, which isn't of our concern.

[2] When $\gamma \geq 1$, it's the interval $[1, \gamma^2]$; otherwise it's $[\gamma^2, 1]$. Note it's r in this interval implying $F(r) \geq \hbar^2/4$, not the other way around.

[3] r is required to be positive for the state to be valid, eq. (26).

because $N \geq n_s > n_d$ and $r \in S$. Therefore by (13), if

$$|2a_2| < |\frac{2b_4 n_d^2 + b_2}{\sqrt{b_4 n_d^4 + b_2 n_d^2 + b_0}}|, \tag{17}$$

then $\partial f / \partial n_d > 0$. We can verify this by squaring and subtraction (also c.f. (12))

$$\begin{aligned} \left(2b_4 n_d^2 + b_2\right)^2 - (2a_2)^2 \left(b_4 n_d^4 + b_2 n_d^2 + b_0\right) &= 4a_2^2(a_0^2 - b_0) \\ &= 16a_2^2 N^2 r \left[(N - n_s) + n_s r\right] \left[r(N - n_s) + n_s \gamma^2\right] > 0, \end{aligned} \tag{18}$$

because $N \geq n_s$. Hence at fixed n_s and N, as n_d increases, the entanglement measure always decreases (14), unless the entanglement measure is zero already.
